# Clinical and psychopathological profile of Tunisian Women victims of domestic violence

**DOI:** 10.1192/j.eurpsy.2022.2229

**Published:** 2022-09-01

**Authors:** R. Jbir, L. Aribi, W. Abid, I. Jbir, W. Bouattour, S. Ellouze, J. Aloulou

**Affiliations:** Hedi chaker, Hospital, Sfax, Tunisia

**Keywords:** Tunisian-women, profile, domestic-violence, clinical

## Abstract

**Introduction:**

Partner violence is a serious public health problem.International studies have well-explored the psychological aspects of domestic violence, but few explored the clinical profile of women victims of violence

**Objectives:**

To define the clinical and psychopathological profile of women victims of domestic violence

**Methods:**

We contacted 75 women who consulted at the psychiatric emergency of ‘HediChaker hospital’Sfax -Tunisia whowhere consulting in the context of medical expertise for domestic violence, on the period between May until October 2021 An anonymous survey was asked to these ladies

**Results:**

The age oscillates between 18 and 64 years 86.7% of the participants were married for the first time, and 24% had at least one child. In 48% of the cases, the victims and their partners had an average socio-economic level. 66.7% don’t have a job. 6.7 % had toxic habits: 5.3% were smoking 22.7% had psychiatric follow: 14.3% for depressive disorder, 7% for bipolar disorder and 1.4% for anxiety Following a physical assault by the spouse, 37.3% of women consulted medical emergency and 21.3% consulted psychiatric emergency. The prevalence of potentially traumatic life events was 29.3%:16% were victim of parental violence and 13.3% suffered from sexual abuse.

**Conclusions:**

This study shows that the prevalenceof domestic violence is higher among young working women. This work underlines also the necessity of a systematic screening of different aspects of violence in emergency medical or psychiatric servicesin order to provide for these ladies the necessary psychological support

**Disclosure:**

No significant relationships.

